# A qualitative study on multisector activities to prevent childhood obesity in the municipality of Seinäjoki, Finland

**DOI:** 10.1186/s12889-022-13658-z

**Published:** 2022-07-06

**Authors:** Leena Koivusilta, Soili Alanne, Marjo Kamila, Timo Ståhl

**Affiliations:** 1 Department of Social Research, Faculty of Social Sciences, 20014 University of Turku, Turku, Finland; 2 The Hospital District of South Ostrobothnia, Seinäjoki Central Hospital, Department of Clinical Nutrition, Seinäjoki, Finland; 3 University Consortium of Seinäjoki, Seinäjoki, Finland; 4 Department of Welfare, Finnish Institute for Health and Welfare, Tampere, Finland

**Keywords:** Childhood obesity, Prevention, Health promotion, Multisector, Municipal

## Abstract

**Background:**

Multisector activities are needed to prevent childhood obesity due to its multifactorial background. The first aim was to identify the activities that had been undertaken for obesity prevention and deduce their main targets. Second, we analyzed the public health policy approaches (upstream, midstream, and downstream) which were followed. Finally, we studied the perception of interviewees regarding their sectors’ roles in implementing the local obesity program.

**Methods:**

Deductive content analysis was used to analyze semi-structured interviews with 34 key professionals (from seven administrative sectors) who had participated in multisector health promotion during 2009–2016 and five representatives of other core parties.

**Results:**

Several midstream and upstream activities were targeted at making physical activity (PA) and healthy eating (HE) integral parts of children’s lifestyle. One long-term strategy was to create environments for PA accessible to every inhabitant and build and renovate the interiors and yards of schools and day-care centers. The healthiness of meals was increased progressively. In addition to midstream and upstream activities, as a downstream activity, an intervention targeting children at risk of obesity was implemented. The impact of management on the activities was considerable; childhood obesity prevention was included in the city strategy and systematically coordinated at the highest managerial level. Altogether, various sectors operated efficiently to promote obesity-preventing lifestyles; however, not all (important) sectors recognized their role in the multisector process.

**Conclusion:**

Most of the activities to guide children towards obesity-preventing lifestyles were either at the midstream or upstream level. Among the latter, considerable work is aimed at creating opportunities to practice PA and making it a natural part of the daily life. The aim of familiarizing children with lifestyles that include PA and HE was shared across sectors, including sectors that had not yet acknowledged their role in obesity prevention. Strong support from city management and systematic coordination of the activity are important factors that contribute to the engagement of several administrative sectors in working towards a shared aim, such as the prevention of childhood obesity.

## Background

Obesity among children threatens human health and reduces life expectancy [1, 2]. In Finland, in 2018, 27% of 2–16-year-old boys and 18% of girls were overweight and 8 and 4% were obese, respectively [3]. Health problems caused by obesity may emerge during childhood [4, 5] and could lead to obesity in adulthood [5, 6].

Risk factors for childhood obesity interact in complex ways and genetic factors also play a role [5, 7]. The web of contributing factors operates at individual-, household-, community-, and wider societal levels [8, 9]. The individual-level factors that most strongly affect obesity risk include diet [10, 11], physical activity (PA) [10, 12], and sedentary behaviors [13, 14]. These lifestyle factors have been targets of preventive programs [8, 15–17].

The environments in which obesity-related behaviors are shaped include family, school, and day-care centers [18–20]. Children’s eating habits are influenced by the type of food available at their homes [21] and their PA depends on parental support [22]. Thus, the lifestyles of families should be targeted in the social contexts in which children live and grow [23–25]. In addition, physical living environments may influence energy intake, behavioral activities, and social norms related to lifestyle [26–28].

There are no simple means to prevent obesity because of its multifactorial background. Obesity prevention programs may be efficient (e.g., [17]); however, policy approaches and widening the spread of related activities in different sectors throughout society could increase the likelihood of longer-lasting effects [29–31]. Based on the knowledge of health determinants [32], public policies and environmental interventions have been found to promote healthy eating (HE) and PA [8, 16].

A framework is required to identify opportunities to prevent obesity at various societal levels. The Obesity Policy Action framework (OPA) by Sacks, Swinburn, and Lawrence [33] is based on a summarized understanding of policy researchers and public health practitioners on policy areas that could be involved. Obesity prevention policies refer to a system of laws, regulatory measures, courses of action, and funding priorities [33]. The framework is theoretically based on the socioecological model which combines the multiple levels on which the various health determinants [32] leading to obesity operate. Thus, the model forms a basis for comprehensive and systematically organized activity aimed at behavior changes among populations and their subgroups [33, 34]. When applying this model, three levels are often recognized. The macro- or society-level includes various public policies aimed at providing opportunities to make healthy lifestyle choices. The interpersonal level covers people’s social and cultural life spheres and communities. The biological and psychological characteristics of individuals play a role on the intrapersonal level. Furthermore, the components of each level interact; for example, personal characteristics may influence the way universally available public services are used [34].

Based on this theoretical model, the OPA framework suggests various instruments that can be used by policymakers to influence the entire population, its subgroups, or individuals. We organized the instruments according to three public health policy approaches. Representing the societal level of the socioecological model, the upstream approach includes policies that influence a wide range of social and economic circumstances, thus creating opportunities for obesity-preventing lifestyles. At the interpersonal or community level of the model, the midstream approach includes ways to influence a population’s behavior in everyday settings. At the intrapersonal level, the downstream approach consists of policy interventions, services, and strategies that offer treatment and support to individuals with obesity or at risk. It is possible to assess the extent to which the implemented activities follow each of these policy approaches by applying the OPA framework to a certain population (e.g., country, municipality). This helps the parties participating in the processes of obesity prevention to integrate policy actions, refine targets, and detect the need for further action [33]. In this study, activities to prevent obesity in the studied municipalities were compared against this model.

In the South Ostrobothnia province, childhood obesity has been a focus of health promotion since the development program for the prevention of type 2 diabetes in 2003–2008. At that time, awareness of obesity risk factors and lifetime consequences was raised among the public, management, and personnel in various administrative sectors [35]. A treatment path called “A Child’s and Adolescent’s Weight Path”, targeting children and parents at risk of obesity, was launched to be used in schools, day-care centers, health care facilities, and family settings [36]. Municipalities, non-governmental organizations (NGOs), and educational institutions participated in this process.

Partly because of this program, the city of Seinäjoki began to focus more on health promotion. Hiring a Director of Health Promotion in 2009 and setting up a health promotion management group, both operating at the city management level, were concrete signs of the beginning of a more systematic approach to the work done across sectors. From 2009 onwards, statistics indicated a significant decrease in the prevalence of overweight and obesity in Seinäjoki. In 2009, 8% of 1-year-olds were overweight or obese; in 2013, the proportion was only 5% and the figures did not increase until 2015. The figures for the 5-year-olds were 17 and 10% in 2009 and 2015, respectively, and did not increase until 2015. In 2011, 14% of 1st-grade students and 16% of 5th-grade students were overweight or obese. In 2013, the proportions were 11 and 16%, respectively; however, in 2015, the figures for the 1st grade and 5th grade students declined to 9 and 8%, respectively [37]. These statistics further boosted obesity prevention activities and emphasized the need to implement multisector cooperation.

We studied the contribution of various administrative sectors, as well as some key organizations, to childhood obesity prevention during 2009–2016. The specific questions were as follows: 1) Which activities did each sector undertake to promote healthy lifestyles, particularly those preventing obesity among children? 2) Which of the main public health policy approaches (upstream, midstream, and downstream) for health promotion and obesity prevention were followed? 3) How did the interviewees perceive their sectors’ roles in implementing the Overcoming Obesity Program?

## Methods

### Setting

The start of the study period was selected as the year 2009, when systematic health promotion started in Seinäjoki. Since our research started in 2016, the study period covered the years 2009–2016. In 2013, the city started participating in the National Obesity Program 2012–2018 [38], titled the Overcoming Obesity Program 2013–2020. Thus, the data collected in this study covered the years before the launch of the municipal program.

### Study design and data collection

The informants were purposively selected by asking the persons coordinating the health promotion activity to name the key professionals in multisector health promotion between 2009 and 2016. The participants represented six administrative sectors. In addition, representatives from three core collaborative partners were interviewed to obtain important information about the process. One researcher contacted the interviewees by phone or e-mail between October and December 2016, inquiring about their interest in being interviewed. In total, 39 people were interviewed; one interview was a group interview with two people and two interviews were obtained as written documents (Table 1). Out of the people contacted, one person refused to participate in the study.

**Table 1:** Administrative sectors and collaborative partners included in the study and the number of interviewees

Administrative sectors and collaborative partners	Administrative areas of the interviewees	Interviews (n)
Management	City managers Political representatives in decision-making	7
Primary health care (maternity and child health clinics, school health care, oral health care)	Maternal and child health clinics, public health nurses Child and school health care, public health nurses Dental health care, dentists Health promotion coordinator	10
Early childhood education and care	Day-care centers Early childhood education	3
Schools	Teachers School principals	6
Sports services	Physical education instructors	2
Technical services	Gardening Municipal engineering Planning manager	3
Mass catering services	Food service managers	3
Hospital District of South Ostrobothnia; The Finnish Institute of Health and Welfare (THL); Representatives of the province	Head of unit Development planners Representatives of province, influential persons	5

The semi-structured interview guide included the following: 1) job description; 2) sector-specific objectives for health promotion and childhood obesity prevention; 3) activities related to health promotion and disease prevention; 4) resources available for health promotion; 5) opinions regarding the visibility and success of health promotion activities and the Overcoming Obesity Program within the interviewee’s sector; and 6) thoughts about the future actions needed. The interviews lasted between 15 and 120 min and were usually conducted in the participant’s office. The interviews were recorded, transcribed verbatim (355 pages), and anonymized. The transcripts were not sent back to the participants for approval.

### Analysis of the interviews

**Fig. 1:**
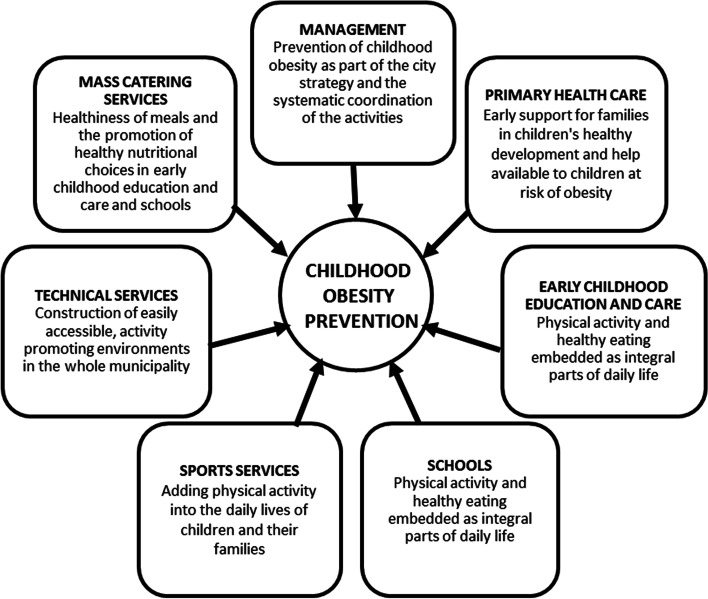
Key activities in childhood obesity prevention in various municipal sectors in Seinäjoki between 2009 and 2016

Deductive content analysis [39] was used to answer the research questions. *First*, three researchers read the transcribed interviews and one of them produced a detailed list of activities undertaken in each administrative sector, as proposed in the National Obesity Prevention Program’s checklist [40] (Table 1). *Second*, three researchers reviewed the findings and formulated shared opinions, which improved the study’s content validity. At this stage, the researchers organized the activities according to the main targets of increasing PA or HE, to support obesity prevention or promote health and/or well-being (Table 2). To summarize the activities, the core (Table 3) and key (Fig. 1) activities were presented for each sector. *Third*, a comparison was performed against the OPA framework [33] to interpret the health policy approaches (upstream, midstream, or downstream) followed in each sector (Table 4). *Fourth,* the responses to the question about the interviews’ perceptions of their sectors’ role in the implementation of the obesity program were analyzed.

**Table 2:** Activities to promote health and prevent obesity and their main targets in the municipal administrative sectors during the period 2009–2016

Administrative sector	Activities according to main targets
Management	*Physical activity* - Investments in PA-promoting environments *Obesity prevention* - Was included in Seinäjoki city strategy - Implementation of the city Overcoming Obesity Program 2013–2020 *Health and/or well-being* - Was included in Seinäjoki city strategy - Director of Health Promotion (2009–2015)^a)^/Health promotion coordinator (2016)^a)^ - Objectives developed in accordance with the National Health Policy Program, and based on the Health in All Policies approach, with special emphasis on children’s health [41] - The mayor and the health promotion management group as supporters of the sector-wise work - Collaboration with NGOs and regular meetings between those working with families with children - Strong commitment of the administrative sectors - Auditing: yearly health overview document, i.e., the Electronic Health and Welfare Report, a nationally developed and obligatory evaluation and planning document [42] to support knowledge management, strategic work, and decision-making in municipalities [43]; surveys among school children - Membership of the Director of Health Promotion in a national health promotion working group led by the Ministry of Health and Social Affairs (support to the work in a municipality)
Primary health care	*Healthy eating* - Dietary advice with dental care *Obesity prevention* - Early intervention and support in healthy lifestyles of children and families - Systematic monitoring of children’s growth - Yearly weight and height reports on the child population - Implementation of the systematically used “Smart Family Method”, which includes a self-assessment tool for families and a motivational interview tool to be used by the professionals [44] - A Child’s and Adolescent’s Weight Path tool for children at risk of obesity [36] *Health and/or well-being* - Preventive services in maternal and child health clinics and in schools - Early support to families in their abilities to promote their children’s healthy development - Systematic monitoring of child health and development during pregnancy (home visits by a nurse) and school age [45] - The “Pilari” [The Pillar] support service for guidance on health and psychosocial problems of children, youth, and families [46]
Early childhood education and care	*Physical activity* - Construction of PA-friendly and inspiring courtyards and interiors, in collaboration with the technical services - Guidance towards including PA as a natural part of the children’s daily life - Introduction of the “Ilo kasvaa liikkuen” [Joy Grows through Motion] program [47] - PA messengers circulating in the day-care centers to distribute information and new ideas - Involvement of families in PA together with their children - Raising awareness about children’s national PA recommendations [48] - Advice given to private childminders *Healthy eating* - Three nutritionally balanced [49] and heart-friendly meals (The Heart Symbol of the Finnish Heart Association (https://www.sydanmerkki.fi/en/)) during full-time childcare - Day-care centers became dessert-free (no sweets, biscuits, potato chips, juice) - Theme weeks for learning HE (e.g., the “Kaappaa kasvis!” [Grab the Veggie!] campaign [50] - Participation in the meal committee together with the mass catering services - Advice given to private childminders *Health and/or well-being* - Comprehensive promotion of the children’s development, overall well-being, and learning [51]
Schools	*Physical activity* - Implementation of the “Finnish Schools on the Move programme” [52] to embed PA as an elementary part of daily life - Construction of PA friendly courtyards and interiors in collaboration with the technical services - Availability of both indoor and outdoor equipment for games and play - Acquisition of new type of furniture (standing desks, etc.) to counteract too much sitting - Breaks from lessons for gymnastics (older pupils trained to act as peer sports instructors) - Restructuring of school days to have a longer break for PA (provided also by agencies outside the school; e.g. the Adult Education Center, congregations) - PA theme days and events (also with families) - PA-weighted curriculum. PA Academy in collaboration with the Kuortane Olympic Training Center (https://epuburheiluakatemia.fi/oppilaitokset/)^b)^ - A teacher as a PA coordinator (one day weekly, in collaboration and the cost shared with the sports services) - Multisectoral teams and teacher collaboration to share ideas - Bicycles provided for students^b)^ - Parents’ association used for communicating about PA opportunities^b)^ *Healthy eating* - Nutritionally balanced [49], heart-friendly meals (The Heart Symbol of the Finnish Heart Association (https://www.sydanmerkki.fi/en/)) - Participation in the meal committee together with the mass catering services - A principle of tasting everything and the observation of portion sizes by the teachers and mass catering personnel - Energy drinks banned in schools *Health and/or well-being* - Restructuring of school days to have a longer break to be used for hobbies (also other than PA, provided also by agencies outside the school, e.g., the Adult Education Center, congregations) - Handbook on well-being available (also describing how to combine PA with cultural experiences) - Nomination of some pupils “responsible for” well-being in classes^b)^ - Adding to the sensitivity of the community for early detection and multiprofessional (teachers, student welfare services, school social workers, school nurses, other primary health care professionals) intervention in students’ problems - Hiring of a professional youth worker called “school coach”
Sports services	*Physical activity* - Investments in sports areas, premises, and activities (playgrounds, winter sports areas, jogging tracks, sports halls, etc.) - Investments in hiring PA instructors and financial support to sports clubs - Swimming schools for children, parents, and other adults - Introduction of new forms of PA - Active communication about opportunities for PA to the public - Adapted and targeted possibilities offered for special groups (e.g., children with asthma) - Collaboration with the Adult Education Center in the organization of PA groups and events - Organization of PA events for children and families - A guidebook prepared for the public on opportunities for PA (and healthy lifestyles) *Health and/or well-being* - A guidebook prepared for the public on opportunities for (PA and) healthy lifestyles
Technical services	*Physical activity* - Planning, building, and maintenance of PA promoting and attractive areas (jogging tracks, parks, playgrounds, schoolyards, etc.) in collaboration with several other sectors and NGOs - Construction of routes for light traffic (walking, cycling, etc.) - Construction of the new plan for the city center to add to PA (and well-being) among the public *Health and/or well-being* - Construction of the new plan for the city center to add to (PA and) well-being among the public
Mass catering services	*Healthy eating* - National nutritional recommendations [49] followed for food preparation at early childhood education centers and schools - The recipes and foodstuff complying with the criteria of The Heart Symbol of the Finnish Heart Association (https://www.sydanmerkki.fi/en/) and the consideration of sugar and fiber contents - Less sugar in snacks, spices to compensate the reduced salt amount - Participation in dessert-free early childhood education and care - Staff training (e.g., Bachelor’s degree in Food and Hospitality) - Feedback collected about meals and observation of meals at schools - Participation in collaborative meal committees

^a)^ The posts were administered in primary health care. The mandate of the Director of Health Promotion was among the highest city management ^b)^ Mentioned in one school only

**Table 3:** Summary of the activities carried out in the municipal administrative sectors according to their main targets

Administrative sector	Main targets of the actions			
	Promotion of physical activity	Promotion of healthy eating	Obesity prevention	Promotion of health and/or well-being
Management	• Investments in PA-promoting environments		• Included in the city strategy • The city obesity program	• Included in the city strategy • Systematic and scientifically informed multisector work led by the Director of Health Promotion and supported by the mayor and the multisector health promotion management group • Strong commitment of the sectors, collaboration with NGOs and those working with families • Matching of the activity with the national level guidelines and the Health in All Policies approach • Auditing of the process of health promotion
Primary health care		• Oral health care professionals giving dietary advice	▪ Systematic monitoring of the children’s weight and height and yearly reports of the entire child population ▪ A specific tool to help children at the risk of obesity	• Systematic procedure to support the early start of healthy lifestyles in maternal and child health clinics and in schools • Systematic monitoring of the children’s development • Support and guidance for health and psychosocial problems
Early child education and care	• The construction and renovation of premises to promote PA • Participation in a national PA program and other activities to help making PA a natural part of daily life • Dissemination of the awareness of the PA recommendations and involvement of the parents in children’s PA	• Nutritionally balanced and heart-healthy meals • Teaching children to learn new tastes and HE • Dessert-free early childhood education and care • Development of the meals in collaboration with the catering services		• The promotion of children’s development, overall well-being, and learning opportunities
Schools	• The construction and renovation of premises to promote PA • Participation in a national program to help making PA a natural part of daily life • Pupils and teachers as PA instructors and innovators • School day restructuring to give space for PA and PA added in the curriculum in some classes • PA events	• Nutritionally balanced and heart-healthy meals • Encouragement in exploring new tastes • The ban on energy drinks • Development of the meals in collaboration with the catering services		• School day structuring to give space for activities which promote well-being • Sensitivity in detecting pupils’ problems and early intervention to address them • Showing ways to add to well-being by combining PA with cultural experiences
Sports services	• Construction of low-threshold, easily accessible, and freely available PA areas and facilities • Hiring of instructors and organizing PA groups and events • Teaching PA skills to both children and parents • Provision of information on PA opportunities and listening to the citizens’ wishes • Targeted PA opportunities for children with special needs			• Provision of information on healthy lifestyles
Technical services	• Collaborative work done with several administrative sectors and NGOs to construct multipurpose and easily accessible areas for PA • The restructuration of the city center to promote PA and the construction of routes for light traffic			• The restructuring of the city center to promote well-being
Mass catering services		• Nutritionally balanced and heart-healthy meals • Meals as a means for the overall promotion of HE • Participation in the meal committees and collection of feedback • Participation in the dessert-free early childhood education and care • Training of the staff

**Table 4:** Main targets of the activities and the public health approach followed in the municipal administrative sectors

Administrative sector	Main target(s) of the activity	Public health approach to obesity prevention		
		Upstream	Midstream	Downstream
Management	Physical activity	X		
	Obesity prevention	X	X	
	Health and/or wellbeing	X		
Primary health care	Physical activity		X	
	Healthy eating		X	
	Obesity prevention	X	X	X
	Health and/or wellbeing		X	
Early childhood education and care	Physical activity		X	
	Healthy eating	X	X	
	Health and/or wellbeing		X	
Schools	Physical activity	X	X	
	Healthy eating		X	
	Health and/or wellbeing		X	
Sports services	Physical activity	X	X	X
Technical services	Physical activity	X	X	
	Health and/or wellbeing	X		
Mass catering services	Healthy eating	X	X	

## Results

### The activities to promote health and prevent childhood obesity according to their main targets

In the detailed list, the activities were organized according to their main targets (Table 2). It should be noted that no activity was part of a specifically funded intervention and was performed as a regular employee duty.

Table 3 summarizes the activities through which each of the four main targets were focused on. The cross-cutting target was to create environments in which PA could be practiced and become a natural part of children’s daily lives. For this purpose, day-care centers and schools built and renovated their premises and equipment was made available for games and play. National level activating programs were participated [47, 52] and even the youngest children and their parents were made aware of the national PA recommendations [48]. In some schools, the pupils and teachers were given roles as PA instructors and innovators. PA-weighted curricula were also used. The restructuring of the school day made longer break for activities, possible. Lectures were allowed to be paused for exercising. Information on opportunities regarding PA was distributed, and parents were encouraged to participate.

Sports and technical services constructed multipurpose and easily accessible premises for PA for all city inhabitants. Longstanding work in planning, building, and environmental maintenance by the technical services was done in collaboration with other sectors and NGOs, and citizens’ wishes were listened to. The restructuring of the city center aimed at creating an environment that would inspire PA and routes for light traffic were also constructed. Sports services hired PA instructors, introduced new forms of PA, and encouraged both children and adults to learn new skills. PA groups and events were organized in collaboration with other organizations and information about these matters was circulated. Targeted strategies ensured equal opportunities in PA for children with special needs.

The target of promoting HE was obvious in the mass catering services responsible for the supply of meals in day-care centers and schools. HE was also an important target in these educational sectors. National nutritional recommendations [49] and the criteria for heart-healthiness were followed for food preparation (https://www.sydanmerkki.fi/en/). The meals were regarded as opportunities for exploring new tastes and learning HE; this was also encouraged by specific campaigns [50]. Furthermore, all day-care centers were declared dessert-free and energy drinks were forbidden in all schools. Meal plans were also developed by committees that had representatives from the mass catering services and educational sectors. The professional skills of catering staff were maintained through continuous training. Important dietary advice to children and parents was provided by professionals in oral care, which is a part of primary health care.

The specific target of obesity prevention was visible at the city management level because it was included in the city strategy; furthermore, there was an obesity program operating in the city. In primary health care, systematic monitoring of the children’s height and weight was performed [45] and reported for the entire child population. A specific tool was used to help children at risk of obesity [36].

Much of the work targeted the overall promotion of health and/or well-being. This target was included in the city strategy at the managerial level, and the work done under the leadership of the Director of Health Promotion was supported by the mayor and the multisector health promotion management group. Scientific evidence and previous experience formed the basis for the activities. The guidelines of the national health promotion programs were followed to develop the structures and objectives of the work. This meant that the Health in All Policies approach was applied [41]. The sectors were strongly committed to the action and collaborated with NGOs. Auditing of the entire process was based on using a health overview document, which is a nationally developed and obligatory evaluation and planning document for municipal knowledge management [42, 43]. In addition, surveys of school children were conducted yearly.

The promotion of health and well-being guided the activity in primary healthcare. This included guidance on practicing healthy behaviors in families early. Support for health and psychosocial problems was also available [46]. Children’s overall well-being was also a target in early childhood education and care [51]. In schools, pupils’ well-being was sensitively observed to address the problems early enough. The aim of promoting the well-being of city inhabitants was a motivator for actions in sports and technical services as well. In addition to providing opportunities for PA, restructuring the city center was intended to add to the attractiveness of the environment, bring people together, and enhance recreation and well-being.

In addition to interviewees from the administrative sectors, representatives from three core collaborative partners expressed their observations about the activities that had been carried out (not included in Tables 2 and 3). The success in obesity prevention was attributed to the systematic coordination and the involvement of many crucial sectors in the work, either in collaboration or independently. Further emphasis on the importance of promoting children’s health among the public and various actors was evident by the way the work was conducted and its results were communicated in the municipality. On a practical level, the nutritionist’s input in the hospital district directly served in obesity prevention. The structures and measures that had been developed before the study period were of great importance.

### Public health policy approaches for health promotion and obesity prevention in various municipal administrative sectors

Individual activities within the sectors were arranged according to the public health policy approach (Table 4). Upstream policies influence a wider scope of social and economic circumstances to affect the determinants of obesity and create environments or settings for healthy lifestyle choices. Midstream policies directly influence the behavior of populations. Downstream policies for obesity prevention represent actions in health services and clinical interventions for individuals [33].

In many sectors, various public health approaches have been integrated into the activities. Several upstream activities have been targeted to provide opportunities for PA. The city management’s support of the long-term work done by the sports and the technical sectors in the creation of environments for PA all over the city and for all inhabitants was important. At the management level, upstream activity was also directly targeted at obesity prevention in the form of the obesity program implemented in the city and by including the aims of obesity prevention and the promotion of health and well-being in the city strategy. The Director of Health Promotion played a key role in leading the activity and bringing together the relevant professionals and other parties to listen to them and disseminate information. All health-promoting work of professionals among their target populations was strongly supported by city management. The primary healthcare sector operated at an upstream level, because one of its tasks is to monitor the health and development of each child, including the measurement of their height and weight [45]. A central upstream operator, mass catering service, is responsible for offering healthy meals to all children. Early childhood education and care further boosted this work by declaring all day-care centers dessert-free.

Midstream activity is practiced in many sectors that aimed to make PA and HE elementary components of the lifestyles of children and their families; however, action was also targeted directly at obesity prevention, in addition to health and/or well-being among children. In primary health care, one core activity is to provide guidance to families and encourage them to follow healthy lifestyles. The role of the downstream approach is minor. It included a service designed to help children at risk of obesity and special services for children who had difficulties in making use of available PA opportunities.

### The perceived role of the overcoming obesity program in the administrative sectors’ action

The managerial level of the city clearly identified its role in obesity prevention, which resulted in the inclusion of obesity prevention in the city strategy and implementation of the obesity program. While the interviewees in primary health care sector were aware of their important work in obesity prevention, they had only gradually become aware of their role in this program. They were convinced about the importance of early intervention, multisector work, and systematic collection of the children’s height and weight data. Both educational sectors identified the importance of their work in obesity prevention. They strongly believed that PA and HE are routes towards obesity prevention. The sports service sector was aware of its role; however, the interviewees representing technical services appeared to be unaware of their sector’s role in the obesity program. Staff members for mass catering were extremely aware of their importance in the obesity program.

## Discussion

A key finding was that the importance of health promotion and obesity prevention was not only rhetorical, but also reflected in concrete actions. In addition, the activities were performed without extra resources as a part of the professionals’ duties. A large part of the work was directed at the most targeted determinants of obesity, PA and HE (e.g., [16, 33]). Children were encouraged to adopt *physically active lifestyles*. Easy-to-access environments and premises for PA were built, and planning was conducted in cross-sector collaboration. For HE, national nutritional guidelines and heart-healthiness were followed to provide *healthy meals* for children in day-care centers and schools. *The prevention of childhood obesity* was included in the city strategy and in the systematic monitoring of children’s growth; specific strategies helped children at risk of obesity. In many sectors, multiple activities have targeted the promotion of *health and/or well-being* among children and all city inhabitants.

The activities were analyzed against the OPA by Sacks et al. [33]. City management operated strongly upstream, creating opportunities and circumstances for the adoption of obesity-preventing lifestyles. Incorporating obesity prevention into the municipal strategy created a supportive environment for action across sectors. The development of the process was based on systematic evaluation [42] and principles of knowledge management [43]. Of crucial importance was the strong leadership of the person responsible for the implementation and coordination of health-promoting activity [53]. Intersectoral collaboration is enhanced if the coordinating person has a sufficiently strong position within the municipal administration [54, 55]. Since this was the case in Seinäjoki, it may have given weight to the director’s initiatives for action and, consequently, strengthened the shared understanding that all sectors’ input was needed (see [56]). The cooperation of the Director of Health Promotion with national programs for health promotion and obesity prevention [40] may have brought new ideas for activities at the local level [57, 58].

The organization of the multiprofessional health promotion activity was dominated by the upstream and midstream levels, with the aim of embedding PA and HE as elementary components of children’s lifestyles. Only two of the reported activities operated downstream. It has been noted that it is important to recognize the interaction between the levels of the socioecological model to fully base health-promoting activities on knowledge about the determinants of health [34]. This helps to create synergies by integrating different public health approaches, sectors, and settings [33, 58, 59]. The interviews revealed synergies and shared targets, particularly in the way the city management, educational sectors, and sports and technical services collaborated to create environments for PA. Additionally, the promotion of HE was a target shared by the mass catering services and educational sectors. Several midstream activities took place in settings in which children leisured and spent a large part of their everyday lives. Furthermore, the work done upstream by management strongly supported the grassroot work for health promotion that the professionals engaged in, midstream.

More information about the prerequisites for the creation of synergies was provided by inquiring about the sectors’ perception of their roles in the implementation of the municipal obesity program. Obesity prevention, as described in the city strategy, stimulated activity in most sectors. In accordance with previous research [26, 60], it was recognized that obesity is not only an individual but also a societal problem that requires collaboration to tackle it [29]. However, in some sectors, obesity programs were not identified. An example was technical services, which were key operators in providing opportunities for PA. A lack of awareness about obesity issues outside the health sector has been observed earlier [61]; it has been said that more efficient collaboration and use of resources would be possible if the awareness of the shared aims also reached sectors which do not have health promotion stated among their objectives [62–64]. Altogether, there seemed to be a good basis for implementing shared goals and further development of the synergies between the sectors; however, work remains to be done to spread awareness in some sectors about the importance of their input for efficient municipal health promotion.

The prevailing principle in taking actions in Seinäjoki was universalism; therefore, all opportunities for obesity-preventing lifestyles were meant to be received by every child. No such measures explicitly considered the economic, political, and social resources of children and their families [32, 33]. However, obesity in children is related to their social background [9, 65, 66] through various mechanisms, such as distress [8, 62] or readiness to make healthy nutritional choices [21, 67]. The professionals’ increased awareness of any source of social inequality could help in planning measures that would motivate various subgroups of children to make more efficient use of the opportunities offered at the upstream level to lead healthy lifestyles. The identification of groups that need special support would enable targeting more downstream activities, specifically to those who would benefit from them the most.

The OPA framework highlights the role of civil society and the private sector in obesity prevention [33]. The private sector was not explicitly mentioned in the interviews; however, its integration into obesity prevention activities could strengthen the work done upstream in many ways. This could take place, for example, by advertising and pricing products. Collaboration with several NGOs was active in the city, which adds to the potential of the facilities to reach various subgroups of the population. In addition, other social operators, such as mass media, could also be engaged in these actions.

Counteractive forces to the activity, such as a conflict between the healthiness of food and its perceived taste, also existed. Despite these controversies, various new forms of action have been created with intense municipality and international collaborations. Thus, the multisectoral activity carried out enthusiastically in one municipality may be a source of inspiration and a model for activity elsewhere, even though municipalities differ according to leadership, resources, and staff training [58]. Due to this and the complex system causing obesity, no clear solution exists for the organization of the action [8] and the menu of activities involved. For example, even within one municipality, individual schools may choose different repertoires. In Seinäjoki, the activity took place in the everyday environments of children and families [24, 25] and many key people in various sectors committed to it. To condense the messages of this study, we suggest that it may have highlighted some key lines of action which could be followed by various administrative sectors in municipalities to prevent obesity among children (Fig. 1).

### Strengths, limitations, and future research

The interviewees represented the administrative sectors and collaborative partners that formed the central operators in health promotion and obesity prevention during the study period. There may be other parties, not included in the study, which would be useful for understanding the entirety of the municipal health promotion activities. The municipal social services should have been included to reveal any sources of inequality or social problems that hinder children and families from making healthy choices. Social service providers were asked to attend but no response was received, which may be because of the high workload in that sector. The interviewees were informed of the interview guide for the first time by the interviewer. A written interview form given in advance could have given the interviewees more time to consider their answers. In addition, questions about interviewees’ formal education or training in obesity prevention would have enabled the assessment of information against professional backgrounds. It would be useful to analyze the extent to which the organization of collaborative networks is based on professions, fields of education, formal collaboration within the governmental system, and friendly relations. Research is also needed on the significance of various governance systems for the organization of strategies to reduce obesity [57] as well as the influence of various health and health promotion concepts on the working methods in obesity prevention in various sectors.

## Conclusion

Obesity prevention was incorporated into the city strategy and systematically coordinated with various sectors strongly engaged in preventive collaboration. Both upstream and midstream public health policy approaches were followed, which meant that opportunities and environments were built and guidance was provided to help children embed PA and HE in their daily lives. Growth and development of the child population were also systematically monitored. The role of the downstream approach was minor but included an individually targeted intervention method for children at risk of obesity and PA opportunities for those with special needs. Altogether, a well-functioning network of highly motivated professionals from many sectors operated for obesity prevention and most of these professionals acknowledged their sectors’ role in this work. However, in some sectors, even if the work done was crucial, professionals did not identify their contributions to the broader work.

## Data Availability

The data that support the findings of this study are available from the corresponding author, [LK], upon reasonable request. The data are in Finnish.

## Abbreviations

PA: Physical activity
HE: Healthy eating
OPA: The Obesity Policy Action framework
NGO: Nongovernmental organisation
